# Effects of gastric sleeve surgery on the serum levels of GH, IGF-1 and IGF-binding protein 2 in healthy obese patients

**DOI:** 10.1186/s12876-020-01309-9

**Published:** 2020-06-25

**Authors:** Khalid Al-Regaiey, Suad Alshubrami, Ibrahim Al-Beeshi, Torki Alnasser, Abdulnasser Alwabel, Hassan Al-Beladi, Omar Al-Tujjar, Abdulrahman Alnasser, Assim A. Alfadda, Muhammad Iqbal

**Affiliations:** 1 Department of Physiology, College of Medicine, King Saud University, Riyadh, Saudi Arabia; 2 Current Address: Director of Academic and Training Affairs Continuous Professional, King Salman Specialist Hospital, Hail, Saudi Arabia; 3 Obesity Research Center, College of Medicine, King Saud University, Riyadh, Saudi Arabia; 4 Department of Medicine, College of Medicine, King Saud University, Riyadh, Saudi Arabia

**Keywords:** Gastric sleeve surgery, Growth hormone, IGF-1, IGFBP-2, Obesity

## Abstract

**Background:**

Bariatric surgery is an effective treatment for severe obesity. It also ameliorates diabetes independently of weight loss through mechanisms that are not fully understood. In this study, we investigated the levels of GH, IGF-1 and IGF-binding protein 2 (IGFBP-2) after gastric sleeve surgery in healthy obese individuals.

**Method:**

This study was conducted in 33 obese (BMI > 38.3) healthy male subjects aged 25 to 50 years undergoing sleeve gastrectomy. GH, IGF-1 and IGFBP-2 levels were evaluated by ELISA at baseline and 6–12 months after surgery. Other parameters, such as glucose, BMI, insulin, HOMA-IR and lipid profile, were also investigated.

**Results:**

Systemic GH (12.32 vs. 50.97 pg/mL, *p* < 0.001) and IGFBP-2 levels (51.86 vs. 68.81 pg/mL, *p* < 0.001) were elevated after bariatric surgery. There was no change in IGF-1 level from before to after surgery. BMI (52.18 vs. 40.11, *p =* 0.001), insulin (19.35 vs. 8.80 mIU/L, *p* < 0.001) and HOMA-IR index (6.48 to 2.52, *p* < 0.001) were reduced after surgery. Lipid profile analysis revealed that total cholesterol (4.26 vs. 5.12 mmol/L, *p* < 0.001) and high-density lipoprotein (HDL) (0.90 to 1.55 mmol/L, *p* < 0.001) were increased, while triglycerides were decreased, after surgery (1.62 vs. 1.05 mmol/L *p* < 0.001). GH, IGF-1, and IGFBP-2 were not correlated with insulin or lipid parameters.

**Conclusions:**

Our study suggests that improved circulating GH and IGFBP-2 levels may mediate the beneficial effects of gastric sleeve surgery in improving insulin sensitivity and reducing insulin demand.

## Background

Obesity remains a continuing global health concern, and its prevalence has doubled since 1980. According to WHO estimates, in 2016, more than 650 million people are obese, and 1.9 billion people are overweight [1]. It is associated with an increased risk of many chronic diseases, including type 2 diabetes (T2D), hypertension, and cardiovascular disease (CVD). Obesity can be defined simply as abnormal or excessive body fat accumulation to such an extent that health may be adversely affected.

Growth hormone (GH) and insulin-like growth factor 1 (IGF-1) have major roles in metabolic regulation, reproduction and ageing. GH is produced by the anterior pituitary gland in response to growth hormone–releasing hormone (GHRH), which is released by the hypothalamus as a normal reflection of multiple features, such as hypoglycaemia, low free fatty acids in the blood, high amino acids, good exercise and sleep [2]. All these features are diminished in adult subjects with high BMI [3]. Following an increase in BMI, GH secretion is reduced, and lipid metabolism is disturbed, leading to increased T2D and cardiovascular disease risk [4]. IGF-1 reflects GH levels and mediates its growth effects, while the metabolic effects of GH, including stimulation of lipolysis and inhibition of insulin signalling in fat and muscle, are induced directly through the GH receptor [5]. IGF-1 in the circulation binds to IGF-binding proteins (IGFBPs). These IGFBPs act as transporter proteins, modulate IGF-1 actions and regulate its clearance [6]. In obesity, non-esterified fatty acids and insulin inhibit IGFBP production, which increases free IGF-1 in circulation [7].

IGFBP-2 is one of the most abundant IGFBPs and is responsible for several cellular processes, such as cell proliferation, migration, and adhesion, which play a significant role in cancer establishment and progression [8, 9]. It is secreted by differentiating preadipocytes. Plasma IGFBP-2 level can be used as a biomarker of insulin sensitivity, as it helps in glucose metabolism by improving insulin sensitivity [10]. Increased IGFBP-2 has had a strong negative association with the risk of T2D and BMI [11, 12]. Lower circulating levels of IGFBP-2 have been linked with an increased risk of developing metabolic syndrome and increased levels of triglyceride-rich particles [13].

Bariatric surgery is an effective treatment for severe obesity that leads to the improvement and remission of many obesity-related comorbidities, sustained weight loss over time, improvement in quality of life and prolonged survival [14, 15]. Bariatric surgery reduces body weight and improves glycaemic control through reduced nutrient intake and malabsorption. However, other mechanisms, such as changes in the secretion and activity of hormones and neurotransmitters involved in appetite, energy expenditure and glucose metabolism, also add to the beneficial effects of bariatric surgery [16, 17].

The underlying mechanisms of how bariatric surgery influences the physiological metabolic process pre- and post-surgery are not fully understood. Therefore, the aim of this study was to assess the activity of the GH/IGF-1 axis and IGFBP-2 levels in obese patients before and 6–12 months after gastric sleeve surgery and their correlations with other anthropometric parameters and lipid profile.

## Methods

### Subjects

This study was conducted in the Departments of Physiology and Surgery, College of Medicine and Obesity Research Centre, King Khalid University Hospital, King Saud University. This study included 33 healthy male subjects aged 25 to 50 years with obesity grades II & III who qualified for laparoscopic sleeve gastrectomy, were not taking medications, did not have severe postoperative complications, and had complete data at the 12-month postoperative follow-up. Informed written consent was signed by all the participants. This study was approved by the Institutional Review Board, College of Medicine, King Saud University, Riyadh, Saudi Arabia (E-17-2652).

### Clinical examinations and blood collection

All patients were clinically examined by a physician, a psychologist and a nutritionist before surgery and attended surgery and nutrition clinics at 3, 6 and 12 months after surgery. The patients were not on medication for kidney, thyroid, or liver disorders and were taking oral vitamins. BMI was recorded at each visit, and patients were classified according to their BMI results. Blood was taken from patients in the fasting state one day before surgery and 6–12 months later. Serum was separated following centrifugation at 1500×g for 10 min and stored at − 80 °C in aliquots within 30 min of collection. Other parameters, such as routine CBC, lipid profile, glucose, insulin, and liver function test results, were retrieved from the hospital files.

### Enzyme-linked Immunosorbent assay (ELISA)

GH, IGF-1 and IGFBP-2 levels were analysed by indirect Simple Step Human ELISA kits (GH Ab190811, IGF-1 Ab100545 and IGFBP-2 Ab100540) following the manufacturer’s instructions (abcam, Cambridge, UK). Briefly, patient samples (33 pre- and 33 post-bariatric surgery) and standards were reacted with specific antibodies coated in the microplates for each protein under investigation and incubated at room temperature (18–25 °C) for 1 h on a plate shaker. Next, the cocktail of antibodies (capture and detector antibodies) was added and incubated as before. One hundred microliters of TMB substrate was added to the microplate and incubated as previously described. The reactions were stopped by adding 100 μl stop solution to each well, and the absorbance was read by a microplate reader (EL 800, BioTek Instruments, USA) at 450 nm.

### Statistical analysis

Data were analysed using SPSS (IBM Corp. Released 2012. IBM SPSS Statistics for Windows, Version 21.0. Armonk, NY: IBM Corp.). Categorical data are expressed as absolute numbers and percentages. Numerical data are expressed as mean, median, standard error of the mean (SEM) and range. Student’s t-test was used to compare the data pre- and post-surgery. A p value < 0.05 was considered statistically significant.

## Results

### Anthropometric and biochemical assessments

Anthropometric data of the 33 obese male patients are shown in Table 1. BMI decreased after bariatric surgery (52.18 ± 9.86 vs. 40.11 ± 8.92 kg/m^2^ [mean ± SEM], 6–12 months after surgery, *p* < 0.001).

**Table 1:** Anthropometric baseline characteristics of the study subjects

Variable	Mean	SEM
Age (years)	35.12	0.249
Height (cm)	170.97	0.005
Weight (kg)	150.16	0.77

Total cholesterol (TC) and high-density lipoprotein (HDL) levels were increased post-bariatric surgery (4.26 ± 0.027 vs. 5.12 ± 0.026 mmol/L and 0.90 ± 0.007 vs. 1.55 ± 0.011 mmol/L, *p* < 0.001, respectively). There was also a significant increase in low-density lipoprotein (LDL) level post-bariatric surgery (2.62 ± 0.026 vs. 2.98 ± 0.022 mmol/L, *p* < 0.031). Triglycerides (TGL) were decreased after bariatric surgery (1.62 ± 0.038 vs. 1.05 ± 0.012 mmol/L, *p* = 0.028, Table 2). Insulin level was decreased from 19.35 ± 0.304 to 8.80 ± 0.181 mIU/L, *p* < 0.001, and the Homeostatic Model Assessment-Insulin resistance (HOMA-IR) index was reduced from 6.48 + 0.164 to 2.52 ± 0.061, *p* < 0.001, Table 2). There was no significant difference in glucose level before vs. after surgery.

**Table 2:** Demographic and clinical measurements before and after surgery

Parameters	Pre-Surgery Mean ± SEM	Post-Surgery Mean ± SEM	*p* value
BMI (kg/m^2^)	52.18 ± 0.299	40.11 ± 0.270	0.001
GLUC (mmol/L)	7.08 ± 0.085	6.27 ± 0.031	0.681
TGL (mmol/L)	1.62 ± 0.038	1.05 ± 0.012	0.028
TC (mmol/L)	4.26 ± 0.027	5.12 ± 0.026	< 0.001
HDL (mmol/L)	0.90 ± 0.007	1.55 ± 0.011	< 0.001
LDL (mmol/L)	2.62 ± 0.026	2.98 ± 0.022	0.031
HOMA-IR index	6.48 ± 0.164	2.52 ± 0.061	< 0.001
Insulin (mIU/L)	19.35 ± 0.304	8.80 ± 0.181	< 0.001

### Analysis of GH, IGF-1 and IGFBP-2 levels in serum before and after surgery

**Fig. 1:**
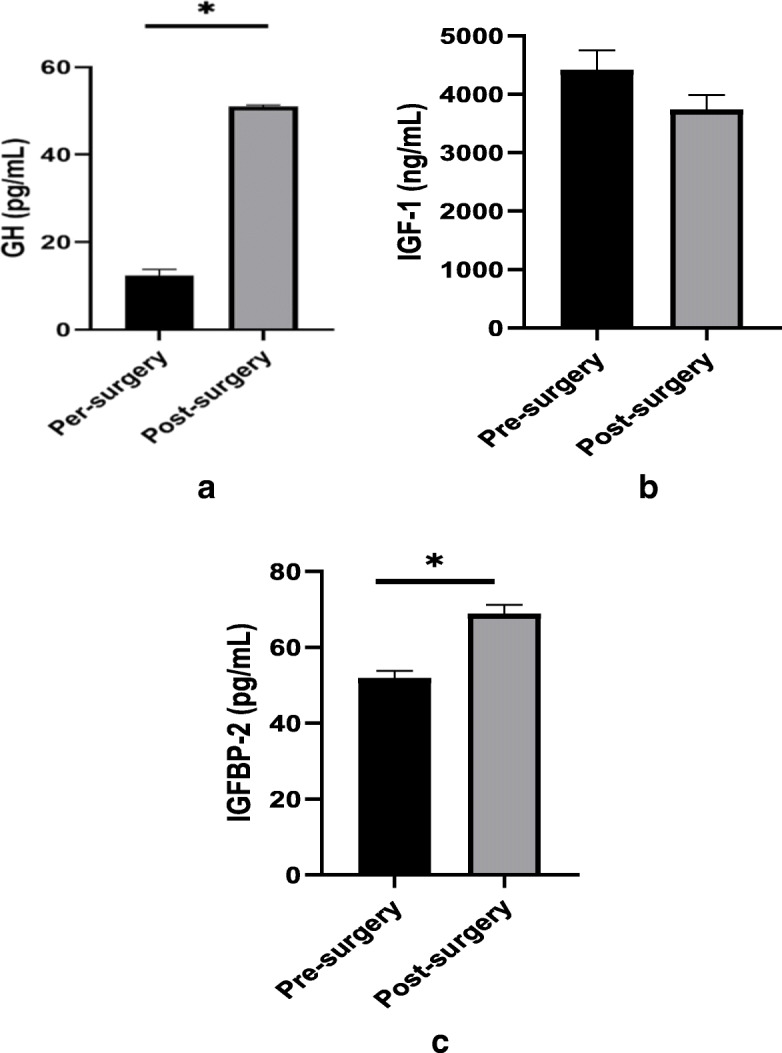
**a-c**. Serum levels of GH, IGF-1 and IGFBP-2 in patients before and after bariatric surgery (*p* < 0.001)

Analysis of the serum level of GH showed an increase post-bariatric surgery (12.32 ± 1.3 vs. 50.97 ± 0.339 pg/mL, *p* < 0.001), while IGF-1 level did not exhibit a significant change before vs. after surgery (4414.38 ± 58.81 vs. 3730.74 ± 43.649 ng/mL, Fig. 1-a and Fig. 1-b). IGFPB-2 increased after surgery (from 51.86 ± 0.34 to 68.81 ± 0.405 pg/mL, *p* < 0.001, Fig. 1-c). There was no correlation between GH, IGF-1, or IGFBP-2 and BMI, insulin or lipid parameters (data not shown).

## Discussion

Bariatric surgical procedures are effective options for long-term obesity treatment to obtain sustained weight loss and substantial improvement of comorbidities and quality of life. Reduced stomach volume and malabsorption are not the only means by which bariatric surgery improves insulin action and associated parameters; alterations in endocrine response are thought to play important roles in the beneficial effects [18]. The aim of this study was to evaluate the effects of gastric sleeve surgery on glycaemic control, lipid profile, GH, IGF-1 and IGFBP-2 among the Saudi adult male population. GH and IGFBP-2 levels were increased after bariatric surgery, while IGF-1 level was not altered. Insulin sensitivity was increased, as reflected by a decrease in insulin level and HOMA-IR index.

The somatotropic axis has an important role in maintaining healthy conditions, and it is suppressed in obesity due to reduced GH and IGF-1 levels in the body. Hyperinsulinaemia and high circulating free fatty acids reduce IGFBP-1 production by the liver, which is responsible for reduced GH production from the pituitary and consequently low IGF-1 levels [19].

IGF-1 has an important role in GH activity and is related to its serum levels. Increased IGF-1 levels in obesity have a negative effect and cause GH suppression [20, 21]. In the current study, GH was elevated after gastric sleeve surgery, while IGF-1 was not changed. Insulin can bind to the IGF-1 receptor, and IGF-1 can bind to the insulin receptor, and both stimulate growth and hypoglycaemic effects. Moreover, hybrid heterodimeric receptors can be formed consisting of an insulin and an IGF-1 receptor α-β dimer, which signal mainly IGF-1 [22]. After bariatric surgery, GH was increased, but IGF-1 was not. It is possible that IGF-1 signalling is enhanced since HOMA-IR was improved. High, low and normal levels of IGF-1 have been reported in obese populations [19, 20]. Our results are in agreement with previous studies reporting no change in IGF levels postoperatively [23, 24]. However, an earlier decrease (3 weeks to 1 month) and an increase 1 year after bariatric surgery have also been reported [25, 26]. In obese children, reduced GH is not associated with decreased levels of IGF-1 or reduced somatic growth [21]. In our study, cholesterol, HDL and LDL were increased after bariatric surgery, but they were still within the desired physiological levels. An increase in the lipid profile can be explained by the lipolytic effects of GH and the release of free fatty acids from visceral adipose tissue and, to a lesser extent, from subcutaneous fat by increasing hormone-sensitive lipase (HSL). Furthermore, GH maintains triglyceride storage in the liver by either inhibiting triglyceride lipolysis via HSL or oxidation by PPARγ [27]. GH also stimulates triglyceride uptake into skeletal muscle to be used for energy or stored as intramyocellular lipids [28]. Furthermore, as a result of bariatric surgery, calorie intake is reduced, which might lead to increased GH levels since its secretion is stimulated by hypoglycaemia. Increased GH after bariatric surgery has beneficial effects on maintaining proper glucose levels and preventing liver steatosis. In the liver, GH stimulates autophagy and preserves plasma glucose levels in chronically starved mice [29]. Moreover, GH signalling in the liver is essential to regulate intrahepatic lipid metabolism, while IGF-1 helps in reducing the catabolic effects of GH [30]. In the current study, although the elevated levels of TC, LDL and HDL may be in part due to increased GH level, there are several potential reasons for this, and not just attributable to increased GH levels.

IGFBP-2 is the main IGF-binding protein associated with regulating body weight and homeostasis and protects against obesity and insulin resistance [10, 31–33]. Obesity-related hyperinsulinaemia increases IGF-1 and inhibits IGFBP-2 secretion [34]. In our study, IGFBP-2 was increased, while insulin level, HOMA-IR index and BMI were reduced 6–12 months after gastric sleeve surgery. IGFBP-2 concentration has been associated with improvements in insulin sensitivity, BMI and lipid profile in obesity-related studies. A recent 20-year longitudinal study of ageing has shown that IGFBP-2 level increases with age, positively correlates with insulin sensitivity, and negatively correlates with BMI at baseline and follow-up [34]. In obese children, circulating levels of IGFBP-2 correlate negatively with body mass and positively with insulin sensitivity [35]. A recent animal study has shown that metformin upregulates IGFBP-2 production through activation of the AMPK-Sirt1-PPARα signalling pathway [36]. Metformin-treated diabetic patients have higher IGFBP-2 levels and lower serum IGF1 levels than untreated patients [36]. This highlights IGFBP-2 as a novel target for metformin action and AMPK-Sirt1-PPARα as a novel pathway to control metabolic syndrome. IGFBP-2 was shown to be increased after biliopancreatic diversion in obese patients and was associated with improved glucose and lipid levels that were sustained even after one year of follow-up [37]. IGFBP-2 has also been shown to be regulated by leptin and may mediate some of leptin’s antidiabetic effects [38]. Early increased levels of IGFBP-2 were noticed after gastric bypass but normalized shortly after [38]. It was recently reported that higher basal levels of IGFBP-2 were associated with lower risk of metabolic syndrome and type 2 diabetes and its levels increased after bariatric surgery [39]. In the current study, the role of IGFBP-2 is suggested to potentially mediate some of the beneficial effects of the sleeve gastrectomy in a healthy male population.

Our study subjects were a small number of homogenous males without medical complications. Future studies should be conducted in a larger sample including women and control lean subjects undergoing non-bariatric laparoscopic surgery for comparison.

## Conclusions

In conclusion, our study suggests that GH and IGFBP-2 levels may be an endocrine response that may in part mediate the beneficial effects of sleeve gastrectomy by improving insulin sensitivity and reducing insulin demand.

## Data Availability

The data used and analysed during the current study are available from the corresponding author on reasonable request.
